# Anti-Glycation Activities of Phenolic Constituents from *Silybum marianum* (Milk Thistle) Flower *in Vitro* and on Human Explants

**DOI:** 10.3390/molecules20033549

**Published:** 2015-02-19

**Authors:** Seoungwoo Shin, Jung-A Lee, Minkyung Kim, Hyunwoo Kum, Eunsun Jung, Deokhoon Park

**Affiliations:** Biospectrum Life Science Institute, Eines Platz 11th FL, 442-13 Sangdaewon Dong, Seoungnam City, Gyunggi Do 462-807, Korea; E-Mails: biost@biospectrum.com (S.S.); biofk@biospectrum.com (J.-A.L.); biotq@biospectrum.com (M.K.); biosd@biospectrum.com (H.K.)

**Keywords:** AGE, fibrillin-1, glycation, human skin explants, silibinin

## Abstract

Glycation is an ageing reaction of naturally occurring sugars with dermal proteins, with clinical signs appearing *in vivo* around age 30, and increasing steadily/regularly with age. The suppleness of the dermis is affected by the formation of bridges between proteins and sugars (Maillard’s reaction). The accumulation of advanced glycation end products (AGEs) in skin plays a very important role in skin ageing. Therefore, natural compounds or extracts that possess antiglycation activities may have great anti-ageing potential. In the present study, *Silybum marianum flower* extract (SMFE) was demonstrated to possess antiglycation activity. We found that SMFE inhibits glycation reaction between BSA and glucose. In addition, antiglycation activity of SMFE was confirmed in a human skin explants model. SMFE reduced N^ε^-(carboxymethyl) lysine (CML) expression, whereas SMFE stimulated fibrillin-1 expression compared to treatment with methyglyoxal. An active ingredient contributing to the observed activities was identified as silibinin. The antiglycation activity of silibinin was dose-dependent. The beneficial effects of silibinin may be applied to prevention or management of AGE-mediated pathologies, targeting in a pleiotropic and complementary way the biochemical and cellular bases of skin aging.

## 1. Introduction

Chronological ageing induces bridging reactions between the amino groups of dermal proteins and the hydroxyl groups of sugars in the skin, *i.e*., Maillard reactions [1]. This translates into a stiffening of linkages between components of the extracellular matrix, which induces, *in vivo*, a decrease in the suppleness of the dermis. *In vivo*, glycation reactions start as soon as reducing sugars come into contact with available free amino groups. The clinical signs of glycation appear when the slowdown of protein expression can no longer counterbalance their degradation rate. Glycation is enhanced by intrinsic factors such as diabetes, or extrinsic factors such as pollution or smoking. Glycation is a reaction of dermal ageing, slow to appear, but producing permanent damages [2,3].

Glycation reactions are characterized by the presence of carboxymethyl-lysine (CML) and pentosidine which are Advanced Glycation End products (AGEs) [4]. AGEs markedly increase in diabetes, due to a higher glycolytic rate, and settle on the elastic network. They arise from dicarbonyl precursors, a category among which methyglyoxal can be included. Among the other markers studied for their sensitivity to glycation, it was shown that fibrillin-1, a glycoprotein associated with oxytalan fibres, is highly sensitive to glycation and its alteration was inversely correlated to the appearance of CML [5]. Fibrillin-1 is contribute to the clinical features of skin aging, such as wrinkle formation and loss of elasticity [6].

Many natural products have shown inhibitory effects on AGEs formation *in vitro* model, but only a few materials were assessed on their efficacy in 3D skin model. The living human skin explants model is well-suited for highlighting the activity of cosmetic products. In this model, glycation can be induced in a few days by methylglyoxal (a reference glycant agent). Aminoguanidine hydrochloride, the reference inhibitor of glycation [7], was added in the culture medium and its efficacy regarding methylglyoxal-induced glycation assessed. Once this model was validated, the efficacy of topically applied formulas containing aminoguanidine HCl or other potential anti-glycation active ingredients against methylglyoxal-induced glycation were studied.

Topically applied vegetable extracts were tested in this model and they were selected on the basis of their reported folk use in the treatment of diabetes and against damage generated by diabetes in some Asian countries [8,9]. Recent scientific studies showed that active ingredients extracted from the “milk thistle” plant (*Silybum marianum*, active ingredient: silymarin) [10,11,12] have a protective activity against glycation, which corroborates the antidiabetic activity of this plant in traditional medicine. Silymarin is able to reduce the level of plasma glycated albumin in diabetic rats and to decrease the content of Advanced Glycation End products. Levels of oxidative and inflammatory biomarkers were also significantly decreased in silymarin-treated groups compared with the diabetic group.

It is therefore interesting and new to test this active ingredient in a skin model in which glycation is induced. Silymarin, a flavonoid complex containing silibinin, silydianin and silychrisin, already has recognized applications in skincare products and their anti-oxidant and protective activities are well known. Silibinin is the major active constituent in silymarin, which is standardized extract of milk thistle plant (*Silybum marianum*). This study aimed to investigate the antiglycation potential of silibinin *in vitro* and in the living human skin explants model.

## 2. Results and Discussion

### 2.1. The Effect of Silybum marianum Flower Extract on AGEs Formation

**Figure 1:**
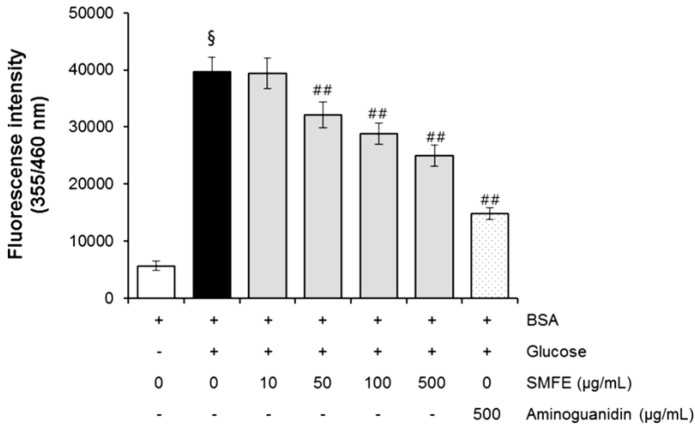
The effects of SMFE on the formation of fluorescent advanced glycation end products (AGEs) in BSA incubated with glucose. Data are mean ± standard deviation of thirty independent experiments. ^§^ *p* < 0.01 compared with the vehicle-treated group, ^##^ *p* < 0.01 compared with the BSA/glucose treated group.

In the present study, for the first time, the AGE inhibitory capacities of *Silybum marianum* flower extract (SMFE) have been evaluated. The BSA-glucose system employed is commonly used in non-enzymatic glycation studies. In the BSA−glucose system, SMFE exhibited significant AGE inhibitory abilities in a dose-dependent manner. As shown in Figure 1, the formation of AGEs was monitored at week 3 by measuring the fluorescence intensity of the BSA-glucose solutions. When BSA was incubated with glucose, a significant increase in fluorescence intensity was observed at week 3 of the experiment. After SMFE was added to the reaction media containing BSA/glucose, the fluorescence intensity significantly decreased in a concentration-dependent manner throughout the study period. At week 3 of incubation, the percentage inhibition of AGEs formation by SMFE (10–500 µg/mL) was 0.6% to 37.1%, respectively.

### 2.2. Topical Anti-Glycation Activity of Silybum marianum Extract

On the third skin explants (female donor aged 34 years old), aminoguanidine and *Silybum marianum* extract were incorporated in a neutral gel at a dose of 1% and applied topically to the surface of the explants for 8 days. After 3 days of treatment, methylglyoxal was added to the culture medium.

**Figure 2:**
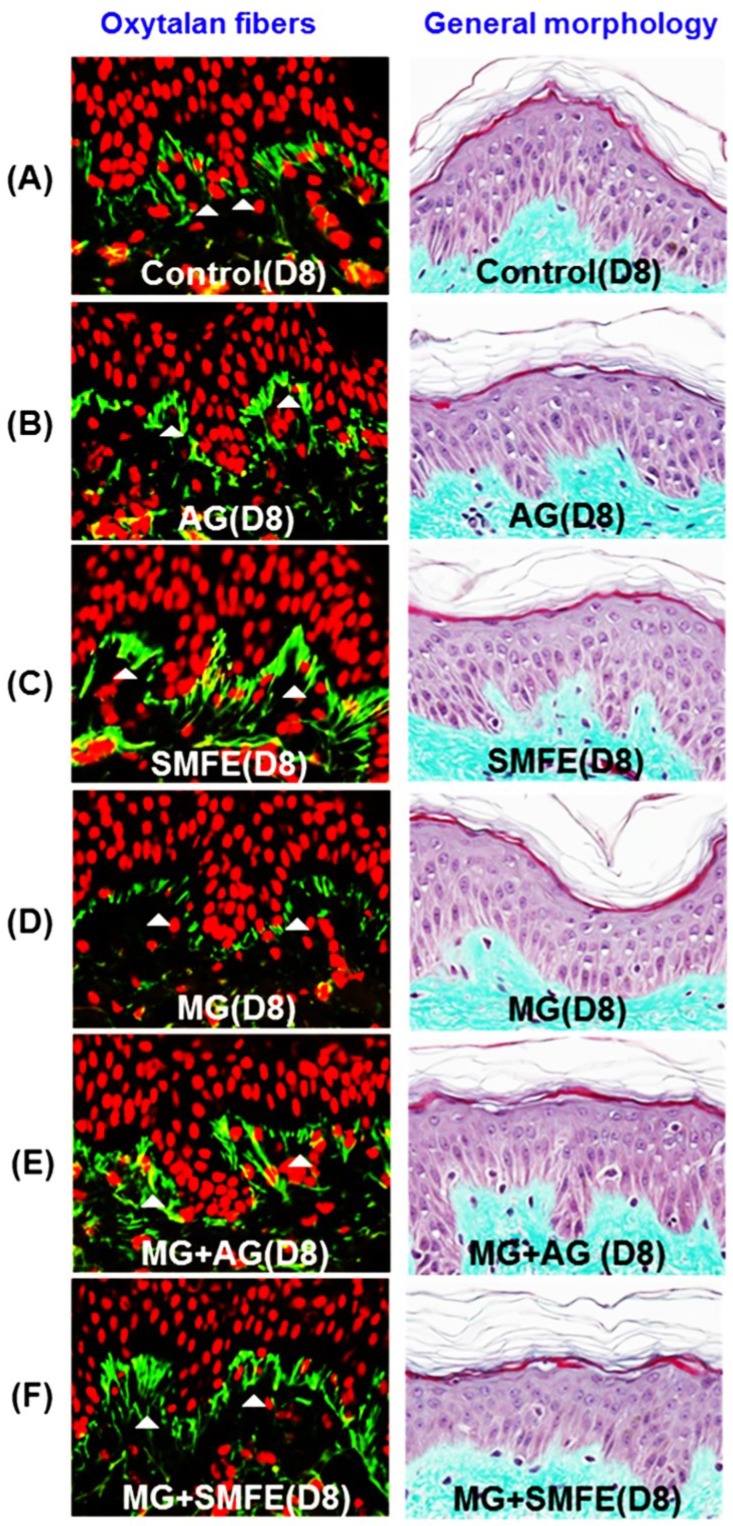

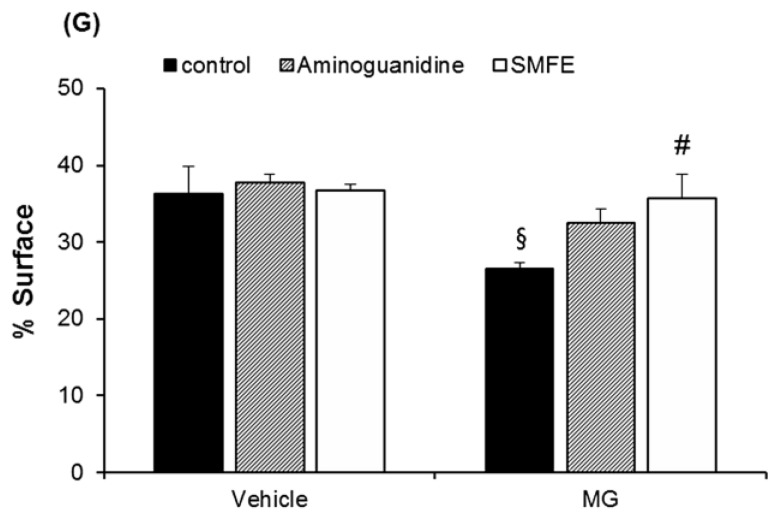
Immunostaining of fibrillin-1 in untreated batch (**A**), treated with AG (**B**), treated with SMFE (**C**), treated with MG (**D**), treated with MG + AG (**E**) and treated with MG + SMFE (**F**). (**G**) Image analysis of the surface percentage occupied by fibrillin-1 under the dermal-epidermal junction, as a function of the product applied. The average fluorescence intensity values were calculated using Image J software. Data are mean ± standard deviation. ^§^ *p* < 0.01 compared with the vehicle-treated group, ^#^ *p* < 0.05 compared with the MG treated group (*n* = 3).

When applied topically to glycated explants, aminoguanidine showed the same protective effect on fibrillin-1 as when added to the culture medium in the second step. The fibrillin-1 network was unchanged compared to the control batch and was protected from methylglyoxal-induced glycation (Figure 2). The degradation of fibrillin-1 through methylglyoxal-induced glycation was clearly highlighted, with a decrease of 26.7% in the surface percentage occupied by fibrillin-1 under the dermal-epidermal junction (DEJ), compared to the untreated control batch. The SMFE titrated into silibinin applied topically also protected fibrillin-1 from glycation and the fibrillin-1 staining was similar to the control batch without induced glycation (Figure 2A–F). These findings were confirmed by the results obtained from the image analysis of fibrillin-1, presented in Figure 2G.

**Figure 3:**
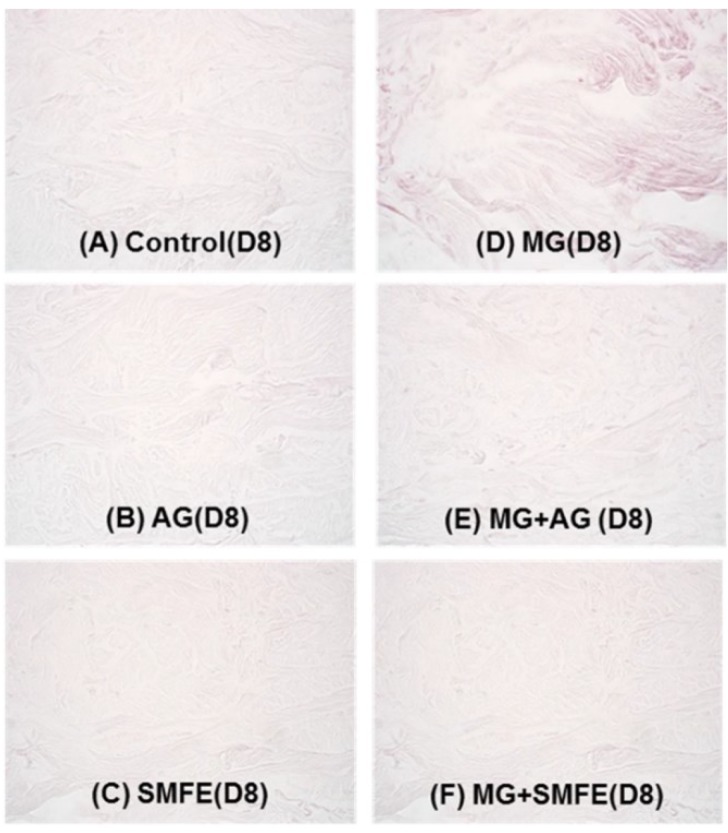

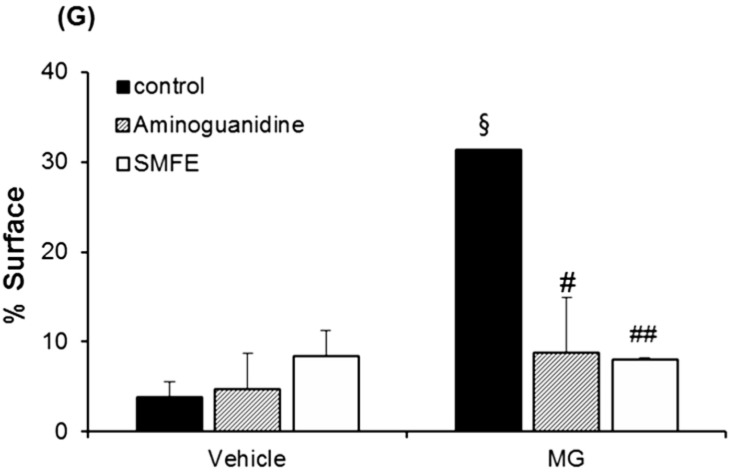
Activity of silibinin against methylglyoxal-induced glycation, revealed by the immunostaining of CML. Immunostaining of CML in untreated batch (**A**), treated with AG (**B**), treated with SMFE (**C**), treated with MG (**D**), treated with MG + AG (**E**) and treated with MG + SMFE (F). (**G**) Staining intensity on batches treated with active ingredients on CML immunostaining. The average staining intensity values were calculated using Image J software. Data are mean ± standard deviation. ^§^ *p* < 0.01 compared with the vehicle-treated group, ^#^ *p* < 0.05 compared with the MG treated group, ^##^ *p* < 0.01 compared with the MG treated group (*n* = 3).

A decrease in the staining of CML (Figure 3A–F) was also observed in the glycated explants treated with AG and SMFE unlike the explants treated with MG. MG induced a clear increase in CML expression on day 8. The SMFE-treated batch (not stimulated with MG) showed complete inhibition of physiological CML expression. The SMFE-treated batch induced by MG showed complete inhibition of CML expression induced by MG. SMFE showed a clear inhibition of CML expression in both the MG-treated and non-MG-treated batches. These findings were confirmed by the results obtained from the image analysis of CML, presented in Figure 3G.

### 2.3. Phytochemical Analysis of Silybum marianum Flower

Our investigation revealed that the content of total phenolic compounds in SMFE was 81.4 ± 2.1 mg gallic acid equivalent/g dried extract. Meanwhile, the content of total flavonoids in SMFE was 0.59 ± 0.02 mg catechin equivalent/g dried extract.

### 2.4. The Chemical Components in the Extract of Silybum marianum Flower

**Figure 4:**
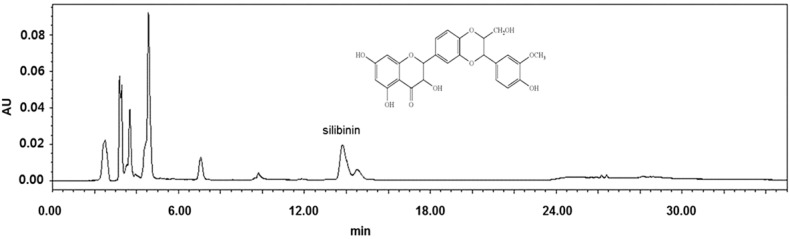
HPLC chromatogram of *Silybum marianum* flower extract at 290 nm.

The chemical components in the extract of *Silybum marianum* *flower* were analyzed by HPLC. As shown in Figure 4, the major peak, tR of 13.824 min, was identified as silibinin (the principal component of silymarin). Its content was calculated as over 0.24% (w/w). To clarify the roles of silibinin, the pure compound was evaluated in antiglycative models.

### 2.5. The Effect of Silibinin on AGEs Formation

**Figure 5:**
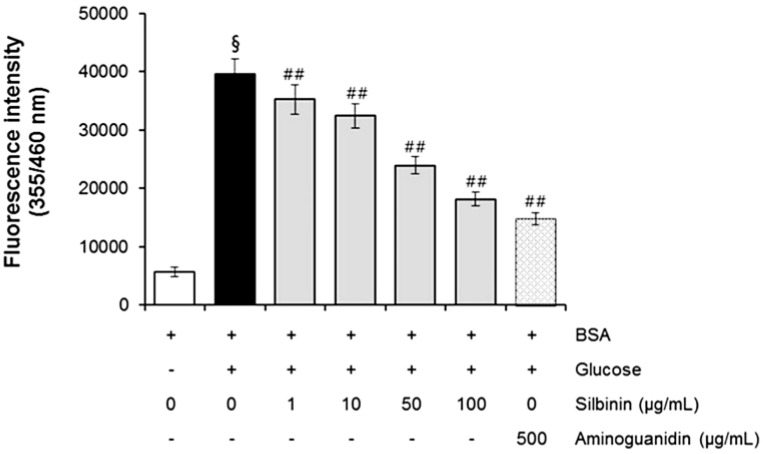
The effects of silibinin on the formation of fluorescent advanced glycation end products (AGEs) in BSA incubated with glucose. Data are mean ± standard deviation. ^§^ *p* < 0.01 compared with the vehicle-treated group, ^##^ *p* < 0.01 compared with the BSA/glucose treated group. (*n* = 3).

In the BSA-glucose system, silibinin exhibited significant AGE inhibitory abilities in a dose-dependent manner. As shown in Figure 5, the formation of AGEs was monitored at week 3 by measuring the fluorescence intensity of the BSA-glucose solutions. When BSA was incubated with glucose, a significant increase in fluorescence intensity was observed at week 3 of the experiment. After silibinin was added to the reaction media containing BSA/glucose, the fluorescence intensity significantly decreased in a concentration-dependent manner throughout the study period. At week 3 of incubation, the percentage inhibition of AGEs formation by silibinin (1–100 µg/mL) was 12.5% to 62.4%, respectively. A significant inhibition of AGEs formation (73.0%) was observed in glucose-induced glycated BSA plus AG (500 µg/mL) (Figure 5). Silibinin showed a strong antiglycation activity (IC_50_ = 70.2 µg/mL), which was 4.8-fold than that of aminoguanidine (IC_50_ = 340.2 µg/mL), a hydrazine-like compound that blocks the formation of advanced glycation end product (AGEs) by amadori-derived products. It is a prototype compound for the prevention of AGEs formation [7]. These results clearly showed that silibinin is an active ingredient contributing to the antiglycative activities of *Silybum marianum* flower.

### 2.6. The Effects of Silibinin on Protein Oxidation

Glycation with glucose rapidly induces protein oxidation and consequently alters the structure of BSA in addition to modifying its biological properties. In particular, the thiol group of Cys residues is especially prone to oxidative attack due to free radical damage to proteins, as it is known that the formation of disulfide bonds occurs during protein aggregation and results in the loss of enzyme activity [13]. The direct oxidation of amino acid (Lys, Arg, Thr) or the secondary reaction of amino acid residues (Cys and His) with reactive carbonyl compounds can lead to the formation of protein carbonyl [14]. Hence, the protein carbonyl content is commonly used as a marker for protein oxidative damage [15].

The determination of carbonyl content was used to assess the protein oxidation that occurred during the process of glycation. As shown in Table 1, the carbonyl content of glycated BSA significantly increased during the experimental period, whereas BSA/glucose incubated with silibinin (1–100 µg/mL) significantly attenuated an increase in the protein carbonyl content of BSA. At week 3, compared to BSA/glucose, the percentage reduction of carbonyl content by silibinin at a concentration of 1–100 µg/mL was 9.9% to 30.2%, whereas the reduction by AG was 21.3%. The present findings suggest that silibinin possesses remarkable potential in reducing protein oxidation.

**Table 1:** The effects of silibinin on carbonyl content in BSA/glucose system.

Experimental Groups	Protein Carbonyl Contents (nmol/mg Protein)	Inhibitory Effect (%)
BSA/glucose	2.31 ± 0.00	
BSA/glucose/silibinin (1 µg/mL)	2.09 ± 0.04 ^b^	9.9 ± 1.7
BSA/glucose/silibinin (10 µg/mL)	1.89 ± 0.03 ^a^	18.3 ± 1.5
BSA/glucose/silibinin (50 µg/mL)	1.79 ± 0.11 ^b^	22.8 ± 4.6
BSA/glucose/silibinin(100 µg/mL)	1.61 ± 0.01 ^a^	30.2 ± 0.4
BSA/glucose/AG (500 µg/mL)	1.82 ± 0.03 ^a^	21.3 ± 1.4

Data are mean ± standard deviation. ^a^ *p* < 0.05 compared with the BSA/glucose treated group, ^b^ *p* < 0.01 compared with the BSA/glucose treated group (*n* = 3).

### 2.7. The Effects of Silibinin on CML Formation

**Figure 6:**
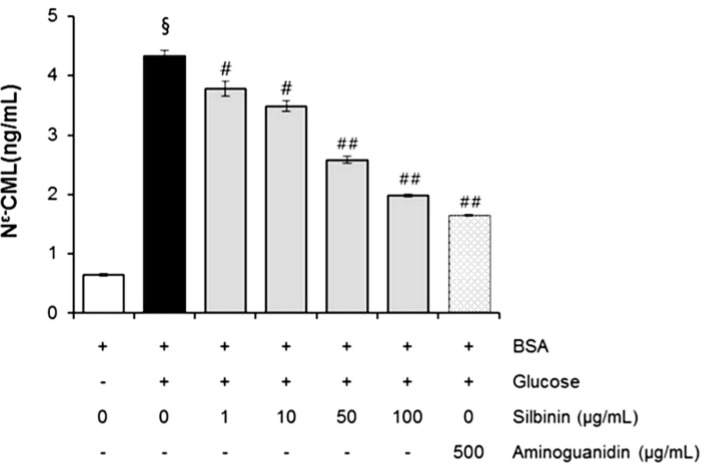
The effects of silibinin on the level of N^ϵ^-(carboxymethyl) lysine (CML) in BSA incubated with glucose after 3 weeks of incubation. Each value represents the mean ± SEM. ^#^ *p* < 0.05 compared to BSA/ glucose; ^##^ *p* < 0.01 when compared to BSA/glucose at week 3 (*n* = 3).

CML has been used as a biomarker for the formation of non-fluorescent AGE. Figure 6 shows that the effect of silibinin on N^ϵ^-CML in glucose-glycated BSA was 6.38-fold higher than in non-glycated BSA. It was interesting to note that silibinin (1–100 µg/mL) significantly reduced the concentration of N^ϵ^-CML in glucose-glycated BSA (12.6% and 54.0%). These results suggest that silibinin can protect against advanced glycation end product formation.

### 2.8. Human Skin Primary Irritation Test of Silibinin

Plant and naturally derived substances may have side effects, including cutaneous irritation and allergic hypersensitivities. Skin primary irritation test in humans (48 h patch test) is general method to determine substance safety. To evaluate the irritation effect of silibinin for clinical applications to human skin, a patch test was performed. Thirty healthy Korean subjects with Fitzpatrick skin type I, II, III were selected on the basis of inclusion and exclusion criteria, and written consent was obtained in each case. The average age was 40.8 years (range 21–47: all females). In our study, as shown in Table 2, none of the 31 subjects experienced a reaction based on the 48 and 72 h readings. Specifically, we did not observe any adverse reactions such as erythema, burning or pruritus in the study subjects that were related to the topical treatment of silibinin. This result suggests that silibinin is safe to use and might be introduced as a possible candidate for topical application.

**Table 2:** Human skin primary irritation test.

No.	Test Material	48 h					72 h					Reaction grade ^b^		
		±	1+	2+	3+	4+	±	1+	2+	3+	4+	48 h	72 h	Mean
1	Squalene	- ^a^	-	-	-	-	-	-	-	-	-	0	0	0
2	Silibinin (0.1%)	-	-	-	-	-	-	-	-	-	-	0	0	0

^a^ No reaction; ^b^ Reaction grade = ∑ [{Grade × No. of Responders}/{4 (Maximum grade) × 30 (Total Subjects)}] × 100 × (1/2).

## 3. Experimental Section

### 3.1. Chemicals and Antibodies

Bovine serum albumin (BSA, fraction V), silibinin, d-glucose, aminoguanidine, and 2,4-dinitro-phenylhydrazine (DNPH) were obtained from Sigma (St. Louis, MO, USA). Trichloroacetic acid (TCA) and guanidine hydrochloride were purchased from Merck (Darmstadt, Germany), respectively. OxiSelect™ Nε-(carboxymethyl) lysine (CML) ELISA kit was purchased from Cell Biolabs (San Diego, CA, USA). All other chemicals used were of analytical grade.

### 3.2. Plant Preparation and Extraction

The flower of *Silybum marianum* was purchased from Jeju (Jeju, Korea). The powdered *Silybum marianum* flower was extracted with 70% ethanol. To prepare the ethanol extract, powdered *Silybum marianum* flower (70.9 g) was extracted overnight with 70% ethanol (5 L) at room temperature with stirring. The supernatant was collected by filtration through filter paper. Ethanol was removed by rotary vacuum evaporation (EYELA, Tokyo, Japan) and the extract was lyophilized (12.2 g).

### 3.3. High Performance Liquid Chromatography

A Waters (Milford, MA, USA) HPLC system with a 600 Controller, 996 Photodiode Array Detector, 616 Quaternary pump, and a 717 Autosampler was used for analysis of silibinin. Data acquisition was achieved using the Waters Empower software. All chromatographic separations were conducted on an ACE^®^ 5 μM C18 column (250 mm × 4.6 mm, 5 μm, ACE, UK) at ambient temperature with detection at 290 nm. The mobile phase consisted of 0.1%trifluoroacetic acid in water and acetonitrile (70:30, *v*/*v*) for 20 min.

### 3.4. Measurement of Total Polyphenolic Content

The content of total polyphenol compounds in the extract was determined using the Folin-Ciocalteu phenol reagent [16]. The content of total polyphenolic compounds was expressed as mg gallic acid equivalents/g dried extract.

### 3.5. Measurement of Total Flavonoid Content

The content of total flavonoid compounds in the extract was determined using a colorimetric assay adapted from Zhishen *et al*. [17]. A total of 150 µL of 5% aqueous AlCl_3_ was added. A total of 1 mL of 1 M NaOH was added 1 min after the addition of aluminum chloride. The absorbance of the solution was measured at 510 nm. The content of total flavonoid compounds was expressed as mg qurcetin equivalents/g dried extract.

### 3.6. In Vitro Glycation of Bovine Serum Albumin (BSA)

The preparation of glycated BSA was performed according to a previously described method with minor modifications [18]. BSA (10 mg/mL) was incubated for 3 weeks at 37 °C with glucose (0.5 M) in 0.1 M phosphate buffer (PBS) at a pH of 7.4. The solution contained 0.02% sodium azide (NaN3) and the incubation was conducted in the absence or presence of silibinin (1–100 µg/mL) and aminoguanidine (500 µg/mL). Aliquots of the reaction mixtures were then assayed for AGEs formation, and CML.

### 3.7. Determination of AGEs Formation

The formation of AGEs was determined spectrofluorometrically (Wallac 1420 Victor3 V, PerkinElmer, Walham, MA, USA) at excitation and emission wavelengths of 355 and 460 nm, respectively. The inhibitory effect of silibinin and aminoguanidine was evaluated by the calculation of percentage inhibition compared with the maximum glycation elicited by glucose. The percentage of fluorescent AGE formation was calculated as follows:

Inhibition of fluorescent AGEs (%) = [(F_C_ − F_CB_) − (F_S_ − F_SB_)/(F_C_ − F_CB_)] × 100 (1)

where F_C_ and F_CB_ were the fluorescent intensity of the control with glucose and the blank of the control without glucose, F_S_ and F_SB_ were the fluorescent intensity of the sample with glucose and the blank of the sample without glucose.

### 3.8. Determination of Protein Carbonyl Content

The carbonyl group in glycated BSA, a marker for protein oxidative damage, was assayed according to a previously published method with minor modifications [19]. Briefly, 400 μL of 10 mM DNPH in 2.5 M HCl was added to 100 μL of glycated samples. After a 1 h incubation in the dark, 500 μL of 20% (*w*/*v*) TCA was added for protein precipitation (5 min on ice) and then the mixture was centrifuged at 10000 *g* for 10 min at 4 °C. The resulting protein pellet was washed three times with 500 μL of ethanol/ethyl acetate (1:1) mixture and resuspended in 250 μL of 6 M guanidine hydrochloride. The absorbance was measured at 370 nm. The carbonyl content of each sample was calculated based on the extinction coefficient for DNPH (ε = 22,000 M^−1^ cm^−1^). The results were expressed as nmol carbonyl/mg protein.

### 3.9. Determination of N^ε^-(carboxymethyl) Lysine (CML)

N^ε^-(carboxymethyl) lysine (CML), a major antigenic AGE structure, was measured using an enzyme linked immunosorbant assay (ELISA) kit according to the manufacturer’s protocol. The absorbance of samples was compared with CML-BSA standard provided in the assay kit.

### 3.10. Human Skin Explants

The first investigations refer to the biological activity of a carboxymethyl cellulose (CMC) gel containing SMFE 1% (silibinin 0.1%) on live human skin explants. The study was performed in accordance with the Declaration of Helsinki after the patient had given informed consent. The explants were obtained as full thickness human skin biopsies from an abdominoplasty of a 34-year-old Caucasian woman. Punch biopsies removed from the patient were immediately sent to Laboratoire BIO-EC (Longjumeau, France), where the hypodermis was removed from the skin and circular samples were excised using a punch instrument. The samples, with the dermis face down, were immediately placed in a liquid-air interface in Laboratoire BIO-EC’s Explant Medium (BEM^®^) and cultured under classical cell-culture conditions (37 °C in 5% CO_2_), and half of the medium (1 mL) was refreshed every other day. The general cells morphology was evaluated prior to all other instrumental observations.

### 3.11. Antiglycation Activity in Human Skin Explants

SMFE was applied on 18 live human skin explants with an average diameter of 11 mm. The preparation of the explants was from an abdominoplasty of a 34-year-old Caucasian woman. The explants were kept alive in BEM culture medium at 37 °C in a humid, 5% CO_2_ atmosphere. The applied product was a carboxymethyl cellulose (CMC) gel with a final concentration of 1% and the application amount was 2 mg/explant. Methylglyoxal (MG) was applied as a glycation promoter at 500 µM on days 3, 5, and 7. Observation of the general morphology was performed after staining paraffinized sections according to Masson’s trichrome method, using the Goldner variant. The immunostaining of fibrillin-1 was performed on frozen sections with an anti-fibrillin-1 antibody, clone 11C1.3 (NB110-8146, Novus Biologicals, Littleton, CO, USA) for 1 h at room temperature with a biotin/streptavidin enhancement system (Vectastain, Vector, Burlingame, CA, USA,) and revealed by fluorescein isothiocyanate (FITC). Nuclei were counterstained with propidium iodide. The immunostaining for carboxymethyl lysine (CML) was performed on a paraffinized section with an anti-CML antibody, clone CMS-10-12 (Trans Genic Inc, Kobe, Japan, ref KH011) for one night at room temperature with a biotin/streptavidin enhancement system (Vectastain, Vector) and revealed by VIP (dark puple) chromagen substrate (SK-4600, Vector).

General morphology, and CML immunostaining were observed using a Leica optical microscope type DMLB at the magnification of 40×. Fibrillin-1 was observed using a fluorescence microscope type DMLB at the magnification of 40×. Photos were taken with a numeric DP72 Olympus camera and stored with the CellD storing software. At least 3 explants were analyzed per each group. The explants were distributed in six batches as follows: Control (D8), no treatment, sampling on day 8; AG (D8), treatment with aminoguanidin 1% solution, sampling on day 8; SMFE (D8), treatment with SMFE 1% solution, sampling on day 8; MG (D8), treatment with MG, sampling on day 8; MG+AG (D8), treatment with both aminoguanidin 1% solution and MG, sampling on day 8, and MG+SMFE (D8), treatment with both SMFE 1% solution and MG, sampling on day 8.

### 3.12. Human Skin Primary Irritation Test

Thirty healthy Korean subjects with Fitzpatrick skin type I, II, III were selected on the basis of inclusion and exclusion criteria, and written consent was obtained in each case. The inclusion and exclusion criteria was defined as follows.

Inclusion criteria

(1) Healthy female, aged from 18 to 60 years old.

(2) Signed and informed consent; the purpose and the protocol of the study were explained to subjects.

(3) Volunteers cooperative and available during the study period.

Exclusion criteria

(1) Volunteers who does not meet the inclusion criteria.

(2) Pregnant, nursing condition or planning to become pregnant within six months.

(3) Participation in previous study without an appropriate intervening period (three months) between studies.

(4) Sensitivity or hypersensitivity skin.

(5) Damaged skin in, or around the test site, which includes sunburn, uneven skin tones, tattoos, scars or other disfiguration on the test site.

(6) Serious renal disorder or hepatic dysfunction.

The average age was 38.1 years (range 24–47: all females). The subjects had no history of allergic contact dermatitis, nor had they used topical or systemic irritant preparations in the previous month. Silibinin (0.1%) formulated with squalene was prepared and applied. The patches (chambers) stayed in place for 48 h. Once the patches were removed, a reading was performed after 48 and 72 h. The readings were rated according to a modified criteria proposed by Frosch and Kligman [20] as well as the Cosmetic, Toiletry, and Fragrance Association (CTFA) guidelines [21] as follows: 0 = no reaction; 1 = slight erythema, spotty of diffuse; 2 = moderate uniform erythema; 3 = intense erythema with ethema; 4 = intense erythema with edema and vesicles. This study was approved by the ethics committee of the DERMAPRO/Skin Research Center, and subjects gave written informed consent.

### 3.13. Statistical Analysis

All data are expressed as mean ± SD. Differences between the control and treatment groups were evaluated by one way ANOVA using SPSS software, version 22.0 (IBM Corporation, New York, NY, USA). A *p* < 0.01 was considered statistically significant.

## 4. Conclusions

Glycated proteins are commonly formed by a non-enzymatic reaction between the interaction of reducing sugars (fructose and glucose) and amino group of protein through a nucleophilic addition with formation of Schiff bases [22]. The unstable Schiff bases further rearrange to produce the formation of reversible Amadori products (such as fructosamine). Subsequently, the Amadori products further form cross-linked structures termed AGEs which can be classified into two major groups: fluorescent and cross-linking structures (pentosidine, crosslines, and imidazolones) and non-fluorescent and non-crosslinking structure (N^ε^-CML).

During the early stage of glycation, Schiff bases are prone to oxidation, generating free radicals, reactive carbonyl groups and the formed AGEs. Consequently, reactive oxygen species-mediated reaction cause the structural fragmentation to create the short-chain carbohydrate intermediates, which then alters sequentially with lysine and arginine residues to produce AGEs [23,24]. Currently, the possible anti-glycation mechanisms have been proposed such as breaking the cross-linking structures in the formed AGEs, blocking the carbonyl or dicarbonyl groups in reducing sugars, Schiff bases of Amadori products, and inhibiting the formation of late-stage Amadori products [25]. In a present study, we investigated the influence of SMFE extract on the formation of total AGEs. The results showed that SMFE efficiently inhibited fluorescent and non-fluorescent AGE formation A significant increase of protein carbonyl content in BSA were seen when the protein was glycated by glucose. When SMFE was added to the same systems, it significantly suppressed these processes. Our findings indicate that SMFE has high content of polyphenolic compounds. Several major mechanisms by which polyphenols block the carbonyl group in reducing sugars and break the cross-linking structure in the formed AGEs have recently been proposed for antiglycation activity [25]. Our findings are consistent with previous literatures indicating that glucose-induced glycation increased protein oxidation as evidenced by increased protein carbonyl content [26]. The present findings suggest that the SMFE has shown remarkable potential reducing the protein carbonyl contents. According to the abovementioned antiglycation mechanisms, SMFE may inhibit AGE formation by scavenging free radicals formed *in vitro* by auto-oxidation of sugars and/or oxidative degradation of Amadori product.

AGEs are considered to be markers of various diseases, such as cataract, arteriosclerosis, renal failure, and Alzheimer disease [27]. Other than glycation, alterations in extracellular matrix content play key roles in aging. As the process of aging advances, extracellular matrix including collagen, elastin and fibers undergo lysis and become thinner. We applied to SMFE on AGEs induced-skin aging model to confirm its potential use for anti-aging ingredient.

Our findings demonstrate that SMFE protects against glucose-mediated glycation *in vitro*. Additionally, SMFE reduces the formation of CML. Furthermore, SMFE clearly prevented glycation on human skin explants.

The model of living human skin explants associated with histology enable researchers to highlight early pharmacological activities, and to visualize in a few days reactions that normally unfold over the long term. Glycation was induced by methylglyoxal over a short period of time with the production of AGEs (CML). Fibrillin-1, a glycoprotein that is a component of a very sensitive part of the elastic network (oxytalan fibres), appears to be a very good marker for glycation activity because it is rapidly altered by MG in this model. SMFE, tested for its potential antiglycation properties, demonstrated that it was able to prevent glycation *ex vivo*. When introduced in culture medium and applied topically, SMFE completely inhibited the glycant properties of methylglyoxal. CML was not observed and the fibrillin-1 network was not altered. An active ingredient contributing to the observed activities was identified as silibinin. The beneficial effects of silibinin may be applied to the prevention or management of AGE-mediated pathologies, targeting in a pleiotropic and complementary way the biochemical and cellular bases of skin aging. Additional investigations are needed to further confirm the observed results as well as to elucidate the molecular mechanisms at the base of their biological activity.
